# The Diagnosis of the Site of the Lesion in Intestinal Obstruction

**Published:** 1907-08-17

**Authors:** 


### THE DIAGNOSIS OF THE SITE OF THE LESION IN INTESTINAL OBSTRUCTION.

In cases of carcinoma of the lower end of the sigmoid and of the first part of the rectum an important aid to diagnosis has been afforded by the invention of the sigmoidoscope. This instrument consists of a metal tube some 25 centimetres long, of such a calibre as to admit of its easy insertion into the bowel by the anus. During introduction the open anterior extremity is guarded by the rounded projection of a well-fitting inner tube. When the instrument is in position the inner tube is withdrawn, and a small electric lamp attached to a battery is introduced, which enables the operator to see the condition of the bowel wall at the end of the instrument. To render the view more clear there is an arrangement by which the bowel can be gently inflated; this prevents the mucous membrane irom clinging round the end of the tube and obscuring the field of vision. The sigmoidoscope has only come into general use during recent years; it provides a means of making an absolutely certain diagnosis in that very common and yet difficult class of case where obstruction exists in the lowest part of the sigmoid or in the upper part of the rectum. These cases are difficult to diagnose for two reasons—the obstruction is too high up for the stricture to be felt by digital examination of the rectum, and too low to be palpated from the abdomen, because it is lying in and protected by the bony walls of the pelvis. Enthusiasts have claimed that the intestine can be examined in this way as far up as the descending colon. This would seem impossible if the anatomical description of the sigmoid (or omega loop, as it is now called) is correct, for it is difficult to see how the instrument could negotiate the very distinct bend found at the lower end of this loop of gut. One word of warning. The instrument must be introduced with great care, especially if the patient is anæsthetised. Any attempt to push the instrument forcibly onwards is dangerous, especially after the removal of the inner tube, because the wall of the intestine is easily ruptured. Two cases of fatal peritonitis, set up in this way, have come to the writer's knowledge.

### Small or Large Intestine?

Apart from these cases where the diagnosis may be made certain, it is generally impossible to do more than to attempt to differentiate stenosis of the small intestine from that of the large. Clinically the signs obtained by examination of the abdomen give the greatest help. In general the position and arrangement of the distended coils of intestine correspond to their normal position. It is, therefore, more important to study their arrangement than their size, and it is a good rule in attempting to identify a particular loop of bowel through the parietes to ascribe it to that portion of the intestinal canal which would normally occupy the position of the loop. The portions most easily identified in this way are the transverse colon and the sigmoid. The coils of small intestine are usually smaller than those of the large gut, but the difference in size is only relative, and should not be relied on too strongly. Distension of the large intestine due to stenosis of the sigmoid most often leads to a fullness of the lateral aspects of the abdomen, and in these cases percussion of the lumbar region will give a resonant note on either side of the vertebral column. The condition may be unilateral from either of two causes. It may be confined to the left side when as yet only the descending colon is distended, or to the right if the stenosis is situated in some part of the transverse colon In stenosis of the small intestine, on the other hand, there is no bulging of the flanks, and the distended coils occupy the centre of the abdomen They are often arranged like a series of tubes tranversely across the abdomen; this has been called the organ-pipe arrangement. All these differences may be completely obscured if the abdominal distension has become excessive.

### Visible Peristalsis.

The character of visible peristaltic movements may aid in the diagnosis of the site of an obstruction. They occasionally stop with definite regularity in one particular part of the abdomen, *e.g.* the iliocæcal valve. When this occurs it may be concluded that the obstruction is in the neighbourhood of the cæcum, especially if the movements resemble small intestine movements—that is, if they are quick and restless. But it must be remembered that if the intestine above the stenosis is fatigued and paretic from overwork and over-distension, the movements may stop at a point which is situated at some distance from the actual seat of obstruction.

Various additional methods have been advocated from time to time as affording assistance in the diagnosis. In particular, elaborate calculations have been made by which the distance of the obstruction from the anus may be deduced from the amount of fluid which the bowel will retain when injected in the form of an enema. Such methods are open to many fallacies, and are, in the writer's opinion, quite futile.

